# Mobile Health Apps for Medical Emergencies: Systematic Review

**DOI:** 10.2196/18513

**Published:** 2020-12-11

**Authors:** Alejandro Plaza Roncero, Gonçalo Marques, Beatriz Sainz-De-Abajo, Francisco Martín-Rodríguez, Carlos del Pozo Vegas, Begonya Garcia-Zapirain, Isabel de la Torre-Díez

**Affiliations:** 1 Department of Signal Theory and Communications, and Telematics Engineering University of Valladolid Valladolid Spain; 2 Polytechnic of Coimbra Escola Superior de Tecnologia e Gestão de Oliveira do Hospital Oliveira do Hospital Portugal; 3 Advanced Clinical Simulation Center, School of Medicine University of Valladolid Valladolid Spain; 4 Emergency Department Hospital Clínico Universitario de Valladolid Valladolid Spain; 5 eVIDA Research Group University of Deusto Bilbao Spain

**Keywords:** mobile health, mHealth, eHealth, Android, iOS, medical emergencies, mobile apps

## Abstract

**Background:**

Mobile health apps are used to improve the quality of health care. These apps are changing the current scenario in health care, and their numbers are increasing.

**Objective:**

We wanted to perform an analysis of the current status of mobile health technologies and apps for medical emergencies. We aimed to synthesize the existing body of knowledge to provide relevant insights for this topic. Moreover, we wanted to identify common threads and gaps to support new challenging, interesting, and relevant research directions.

**Methods:**

We reviewed the main relevant papers and apps available in the literature. The Preferred Reporting Items for Systematic Reviews and Meta-Analyses (PRISMA) methodology was used in this review. The search criteria were adopted using systematic methods to select papers and apps. On one hand, a bibliographic review was carried out in different search databases to collect papers related to each application in the health emergency field using defined criteria. On the other hand, a review of mobile apps in two virtual storage platforms (Google Play Store and Apple App Store) was carried out. The Google Play Store and Apple App Store are related to the Android and iOS operating systems, respectively.

**Results:**

In the literature review, 28 papers in the field of medical emergency were included. These studies were collected and selected according to established criteria. Moreover, we proposed a taxonomy using six groups of applications. In total, 324 mobile apps were found, with 192 identified in the Google Play Store and 132 identified in the Apple App Store.

**Conclusions:**

We found that all apps in the Google Play Store were free, and 73 apps in the Apple App Store were paid, with the price ranging from US $0.89 to US $5.99. Moreover, 39% (11/28) of the included studies were related to warning systems for emergency services and 21% (6/28) were associated with disaster management apps.

## Introduction

Internet and mobile computing technologies have changed people’s lifestyle. With regard to mobile devices in health, mobile devices, such as personal digital assistant devices (PDAs), smartphones, and tablets, have been widely adopted by medical professionals. These devices are quickly becoming some of the main instruments for accessing clinical information, especially for young health professionals and students [1]. Several medical resources are available on the digital distribution platforms of mobile apps (Google Play Store and Apple App Store) for Android and iOS operating systems [2].

According to the World Health Organization, the development of apps for the health domain is directly or indirectly intended to maintain or improve healthy behaviors, quality of life, and people’s well-being [3].

Mobile health (mHealth) refers to the practice of medicine and public health. Robert Istepanian mentioned “the emerging use of mobile communications and network technologies for health” [4]. The field of mHealth has become a subbranch of eHealth, which has to do with the use of information and communication technologies, such as computers, mobile phones, GPS, and patient monitors for health and information services. mHealth includes the use of mobile devices in the collection, delivery, and access of health information by professionals, researchers, and patients. It is an emerging and rapidly developing field, which plays a vital role in the transformation of health care to increase its quality and efficiency.

On one hand, mobile apps are specifically aimed at helping people in their own health and wellness management. On the other hand, numerous mobile apps aim to assist health care providers as tools to improve and facilitate the provision of patient care [5]. According to a recent 2019 report on global mHealth, the market can be segmented based on the following: (1) equipment/connected medical devices, (2) mHealth services, and (3) mHealth apps [6].

The main objective of this paper was to present a systematic review that addresses the study of mobile apps for health emergencies. Furthermore, this paper presents the mobile apps available for the Android and iOS operating systems [7-11]. The main contribution is synthesis of the existing body of knowledge to provide relevant insights and to identify common threads and gaps to support new challenging, interesting, and relevant research directions.

In summary, mobile apps in the health sector are continually growing, and soon, they will be able to change the concept of medicine [7]. These apps will allow patients to access their health information, have small consultations for specific issues without consulting a professional, and locate emergency services. They will also help monitor chronic patients, increase safety in taking medication, and help network with people in the same situation. Moreover, professionals have access to specific information and tools to create new relationships with patients. Prehospital medical care starts from the occurrence of the event, involves transfer, and ends at admission to the welfare institution. Moreover, it always has to be offered by a health care professional. Consequently, we consider these apps as medical emergency apps.

A search for the term “medical emergency apps” in mHealth does not provide results. All reports and studies are focused on mHealth and do not distinguish between the different branches into which this technology can be divided. Therefore, we found a lack of interest in this important domain that can improve prehospital care for patients with the use of new technologies.

The use of mobile apps can facilitate the exchange of information between health professionals in the case of a possible health emergency. Consequently, this analysis has two focuses. First, we review the current status in the literature. Second, we review the mobile apps available in the main virtual stores. Currently, owing to the proliferation of mobile apps in this domain, it is necessary to evaluate their importance to promote health care.

Following the selection of relevant studies, a statistical analysis was carried out. The results have been discussed to analyze the main contributions of each publication. Finally, the most important findings have been reported.

## Methods

### Overview

In this section, the methodology used in this study is defined. The search process for information extraction from the available apps in the field of health emergencies is also reported. Our study focused first on the available literature and second on the available apps in the field of health emergency.

### Literature Review

The procedure for the selection of articles was the same as that followed in other previous work [8]. The articles are analyzed by reading the title and abstract to identify the most relevant papers. The number of scientific publications is very high. Therefore, we followed a protocol that allowed us to synthesize the most relevant information. Two relevant protocols for systematic reviews are Quality of Reporting of Meta-Analysis (QUOROM) [9] and Preferred Reporting Items for Systematic Reviews and Meta-Analyses (PRISMA) [10].

On one hand, QUOROM focuses on the presentation of a meta-analysis of randomized clinical trials and includes checklists for authors, reviewers, and editors of biomedical journals, as well as a diagram of the flow that describes the whole process. On the other hand, the PRISMA protocol is an extension of QUOROM with more pedagogical purposes accompanying the checklist with extensive documentation that justifies a series of check items. Furthermore, PRISMA is applicable to all types of systematic reviews and is not limited to a meta-analysis of clinical trials. This type of protocol was introduced with the idea that clinical trial publications follow the type of standards set by each protocol, and thus, works of this type will be standardized.

Therefore, this study adopted the PRISMA methodology. This process is divided into the following four phases:

- Identification: The title is considered in the choice.
- Selection: The summary for the choice of the paper is taken into account.
- Eligibility: The content is taken into account for the choice.
- Inclusion: We finally obtain the papers with the highest potential content.

The PRISMA protocol starts with the identification phase. In this stage, we used specified keywords to identify the relevant papers in several databases. Using these series of papers, we performed a set of steps to finally obtain the papers with which we carried out our review.

The search engines on which we obtained the different papers for the analysis were as follows: IEEE Xplore, Science Direct, PubMed, Web of Science, and Google Scholar. These databases were used since they cover the majority of papers that are within the scope of this review and include the most relevant sources. Moreover, the above-mentioned databases have been used in several systematic review papers on mHealth [8,11,12]. The papers were selected and screened by two different reviewers. Moreover, all the selected papers were included with the common agreement of all the authors.

The logic and keywords used to conduct this review were as follows: “emergency” AND “app,” “emergency” AND “mHealth,” “emergency” AND “eHealth,” and “eEmergency.”

We focused on review papers and research papers, excluding other results that these search engines offer, such as book chapters, patents, conference summaries, and news. The search was carried out starting from the year 2009, considering the papers published for 10 years until the end of 2019. The search and selection of papers were conducted during March 2020.

Review papers were included since they collect information from the most relevant sources. These papers provide clear and concise insights, which were used to carry out our analysis. Concerning the dates of publication, this systematic review considered the previous 10 years. This review only included papers in the English language because it is the universal language par excellence (Lingua Franca).

**Figure 1:**
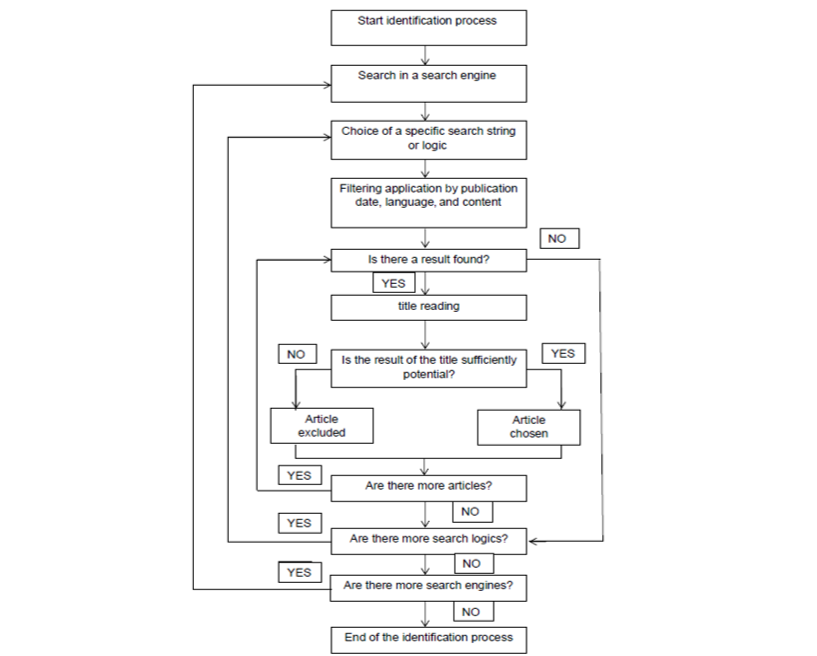
Flow diagram for the identification phase.

After the identification process, the papers were ordered according to relevance. Moreover, the identification process followed the PRISMA protocol that can be represented in a flow chart. Figure 1 presents the section process of a particular search engine through which the search was performed and for choosing a particular search string or logic.

**Figure 2:**
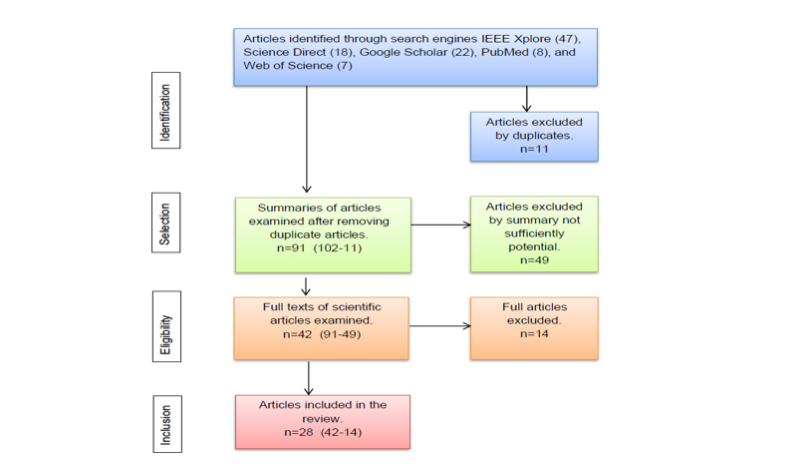
PRISMA flow chart.

After the identification process, the obtained papers from a different database were exanimated to find duplicates. Thereafter, we conducted the selection phase. The section state started with the exclusion of papers by reading the title and abstract. The eligibility phase included reading the full text of the remaining papers obtained in the selection phase. Finally, in the inclusion phase, the final number of papers included in the review were defined. This entire process is presented in Figure 2. In total, we found 28 relevant papers that met the search criteria and respected the exclusion criteria.

### Review of the Apps Available in Mobile Market Stores

After reviewing the available literature, a review of the mobile apps and websites available was conducted.

On one hand, the Google Play Store and Apple App Store were used to search for mobile apps, since they are the two app stores that have more apps. On the other hand, Google Chrome was used to perform a webpage search on eHealth. The search process on available mobile apps was conducted during March 2020.

**Figure 3:**
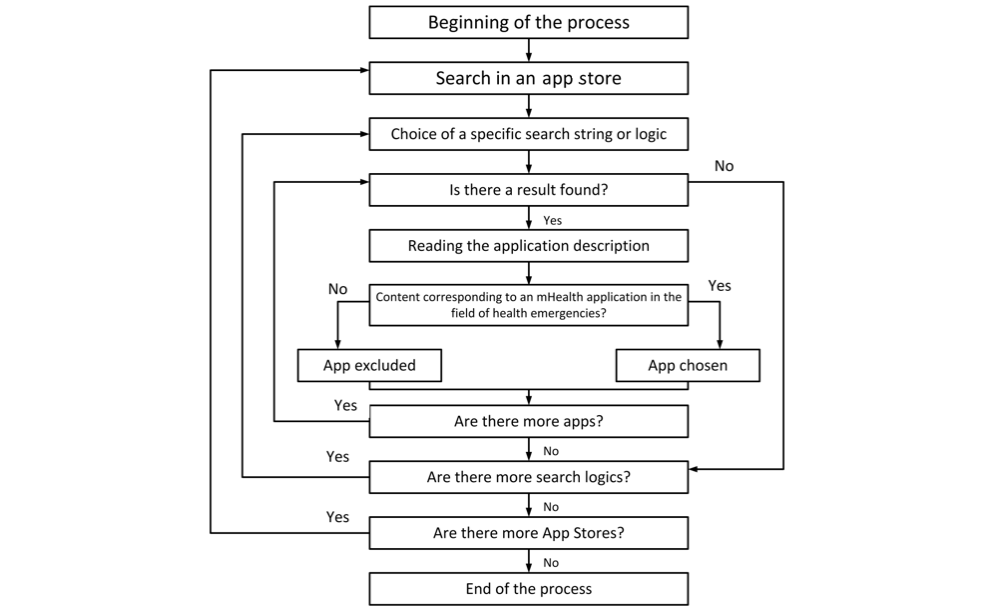
Flowchart process for selection of apps.

The methodology used for this second part was very similar to the flow chart in Figure 3. The following keywords were defined to try to obtain all possible results for this analysis: “emergency” OR “emergencia,” “eEmergency,” “safety” OR “seguridad,” “alert” OR “alerta,” “disaster” OR “desastre,” “SOS,” “112,” and “blood donation” OR “donación de sangre.”

In addition, the language and country used by the different mobile apps were not taken into account. The criterion followed to choose mobile apps in terms of content was to analyze the information offered by each search engine about the app. If the app corresponded to the field of health emergency (whether designed to help health care staff or the patient), it was considered in our study.

Figure 3 presents the chart that was followed in the process of selecting the different mobile apps in our study.

## Results

### Overview

This section presents first an analysis of the results obtained in the literature review and second an analysis of the available mobile apps.

The number of results obtained per year after the analysis and selection conducted by the authors is presented in Table 1. The findings have been categorized according to the year of publication and the number of obtained results.

The distribution of the number of papers selected after the systematic review according to the year is presented in Table 2. The studies analyzed in this systematic review were distributed from 2013 to 2019, and most of the results (n=8) involved 2017.

Table 3 presents the title, the date of publication, and the summary of the main contributions of each paper included in the systematic review [13-40].

**Table 1:** Distribution of publications per year before applying the selection criteria.

Year	Number of studies
2009	21,782
2010	24,995
2011	29,327
2012	33,234
2013	37,770
2014	44,582
2015	51,111
2016	56,082
2017	63,540
2018	67,289
2019	54,735

**Table 2:** Distribution of publications per year after applying the methodology.

Year	Number of studies
2009	0
2010	0
2011	0
2012	0
2013	1
2014	3
2015	3
2016	4
2017	8
2018	7
2019	2

**Table 3:** Main contributions of each paper included.

Title	Year	Main contributions
With the Proliferation of Mobile Medical Apps, Which Ones Work Best in the Emergency Department? [13]	2015	Study on the best apps for the emergency department.
Prehospital emergency notification system [14]	2016	Proposal of a mobile app that allows emergency services to provide the hospital with information about the severity of a victim.
Vehicle-Assist Resilient Information and Network System for Disaster Management [15]	2017	Development of a network for disaster management that will be used if the internet is not available and which is made up of possible drones that collect information from a mobile app and send it to geo-distributed servers.
MHealth based ubiquitous fall detection for elderly [16]	2017	Development of a prototype based on an accelerometer sensor for the detection of falls in the elderly. This prototype uses a mobile app to send an alert to emergency services in case of a fall.
STLS: Smart traffic lights system for emergency response vehicles [17]	2019	Android app for monitoring the traffic network of a city to allow emergency vehicles to circulate without conflicts.
State of the Earthquake Field Disaster Investigation Information Service System [18]	2019	Disaster notification system to inform the inhabitants of a country about the arrival of an earthquake to better manage this type of disaster.
Smartphones let surgeons know WhatsApp: an analysis of communication in emergency surgical teams [19]	2015	Study on the improvement of communication and effectiveness in a surgical team thanks to the use of the instant messenger WhatsApp.
MHealth: Blood donation application using android smartphone [20]	2016	App to make blood donations using mobile devices with the Android operating system.
SaveMe: A crime deterrent personal safety android app with a Bluetooth connected hardware switch [21]	2019	Switch connected to a smartphone via Bluetooth that can be pressed for warning about danger to the emergency contact of the victim.
Click away emergency aid scheme by means of intelligent situation assessment [22]	2018	Development of a mobile app to help people access emergency services easily.
Blood donation and life saver app [23]	2018	App for the search of a blood donor with specific characteristics. Obtain possible donors through geolocation and send them an alert message.
Blood bank app using Raspberry PI [24]	2018	Development of an app using Raspberry to shorten blood donation times.
An effective support system of emergency medical services with tablet computers [25]	2015	Study for the evaluation of a system of tablets installed in emergency vehicles to shorten service times and thus improve prehospital medical care.
An Integrated mHealth and Vehicular Sensor Based Alarm System Emergency Alarm Notification System for Long Distance Drivers using Smart Devices and Cloud Networks [26]	2019	App for monitoring the vital signs of professionals who are dedicated to covering long distances on roads to avoid possible traffic accidents.
Intelligent crash detection and emergency communication system for two-wheelers [27]	2019	Accident detection system for two-wheeled vehicles that warn the driver of critical points where an accident can occur through the use of machine learning technology.
Integration of Emergency Web App for Accessing the Emergency Services by Mobile Phones [28]	2013	App for emergency management that can notify emergency services through a smartphone.
Instantaneous feedback pedometer with emergency GPS tracker [29]	2019	App for notifying emergency services in case of sudden heart failure among people who play sports.
Information and communication technologies for enhanced emergency management in Taiwan high-speed rail [30]	2016	System of identification of an environmental hazard integrated into high-speed trains to improve safety.
iEMS1669: An innovative Med Alert App for Thai Emergency Medical System [31]	2017	App to provide a medical alert to emergency services, family, and friends.
Facilitating the collection and dissemination of patient care information for emergency medical personnel [32]	2016	System for sending information from emergency technicians to the hospital to notify about the patient’s situation and improve prehospital medical care.
Indoor Localization for Evacuation Management in Emergency Scenarios [33]	2018	System formed by an app and a central monitoring system for the correct evacuation of a building.
An Analysis of WhatsApp Usage for Communication Between Consulting and Emergency Physicians [34]	2016	Study on the use of WhatsApp for communication between the administration of consultations and emergency doctors.
A triggering mechanism for end-to-end IoT eHealth system with connected ambulance vehicles [35]	2018	User monitoring system (generally designed for the elderly) to notify an ambulance in case of emergency.
A Mobile/Cloud Emergency Response Application for Indoor Assisted Living [36]	2014	App for notifying emergency services in case of emergency.
A mobile-based emergency reporting application for the Philippine National Police Emergency Hotline 911: A case for the development of i911 [37]	2018	App for the Philippine National Police to accelerate the response time with the collection of user data.
DETSApp: An App for Disaster Event Tweets Summarization using Images Posted on Twitter [38]	2018	App to summarize a disaster by compiling a Twitter post.
Design and development of a crowdsourcing mobile app for disaster response [39]	2017	App to disseminate geographic information in case of an emergency.
Reducing Traffic Congestion Using Geo-fence Technology: Application for Emergency Car [40]	2014	App for monitoring the traffic network of a city to allow emergency vehicles to circulate without conflict.

### Results for the Google Play Store

The results obtained for the Android operating system were analyzed. The categorization of apps provided by the Google Play Store is presented in Table 4.

The results of the apps obtained for Android according to the filter used in the search conducted are presented in Table 5.

**Table 4:** Categorization of apps for Android.

Categorization	Number of apps
Health and Wellness	56
Medicine	48
Communication	17
Tools	17
Social	15
Lifestyle	10
Travel and Guides	6
Education	5
Business	5
News and Magazines	5
Productivity	4
Maps and Navigation	3
Entertainment	1

**Table 5:** Categorization of apps by filter for Android.

Categorization	Number of apps
Blood donation OR donación de sangre	73
Emergency OR emergencia	51
SOS	24
112	11
Alert OR alerta	11
Safety OR seguridad	11
eEmergency	8
Disaster OR desastre	3

### Results for the Apple App Store

The results obtained for the iOS mobile operating system were analyzed. The categorization of apps provided by the Apple App Store is presented in Table 6.

The results of the apps obtained for iOS according to the filter used in the search conducted are presented in Table 7.

**Table 6:** Categorization of apps for iOS.

Categorization	Number of apps
Medicine	20
Tools	19
Health and Wellness	17
Travel and Guides	17
Lifestyle	16
Maps and Navigation	10
Business	9
News and Magazines	9
Education	6
Productivity	4
Entertainment	3
Communication	1
Social	1

**Table 7:** Categorization of apps by filter for iOS.

Categorization	Number of apps
Emergency OR emergencia	38
SOS	32
Alert OR alerta	23
112	13
Safety OR seguridad	12
Disaster OR desastre	7
Blood donation OR donación de sangre	6
eEmergency	1

### Comparison of the Results

Comparison of the categorization of the apps on each platform (Google Play Store and Apple App Store) was conducted, and the findings are presented in Table 8.

The results of all apps obtained according to the filter used are presented in Table 9.

In total, 192 selected mobile apps with potential content were obtained from the Google Play Store, while 132 were obtained from the Apple App Store.

On the Android operating system, the most number of results were obtained in the Health and Wellness, and Medicine categories. Out of the 192 apps obtained, 56 apps belonged to the Health and Wellness category. Moreover, 48 apps belonged to the Medicine category. However, on the iOS operating system, in the Apple App Store, we found a more equitable distribution of the number of apps when considering the category. Out of 132 apps, 20 belonged to the category of Medicine, 19 belonged to the category of Tools, 17 belonged to the categories of Health and Wellness, and Travel and Guides, and 16 belonged to the category of Lifestyle.

In total, 73 and 51 apps were obtained from the Google Play Store using the filters “blood donation” OR “donación de sangre” and “emergency” OR “emergencia,” respectively. For the Apple App Store, 38, 32, and 23 mobile apps were obtained with the filters “emergency” OR “emergencia,” “SOS,” and “alert” OR “alerta,” respectively.

Out of 324 apps, 73 and 68 were from the Health and Wellness, and Medicine categories, respectively.

Finally, regarding the categorization according to the filter applied in the search, the three filters through which more apps with potential content were obtained included “emergency” OR “emergencia,” “blood donation” OR “donación de sangre,” and “SOS,” with 89, 79, and 56 results, respectively.

Regarding the price of apps, we found that all identified apps available in the Google Play Store were free. However, 59 mobile apps in the Apple App Store were paid, with the price ranging from US $0.89 to US $5.99. The price of apps is relevant to their use since it can be a critical limitation to their use by people with economic issues.

In total, 79% (22/28) of studies were found in the IEEE Xplore database. Moreover, 17% (5/28) of the studies were selected from Google Scholar and 4% (1/28) from ScienceDirect. Finally, an analysis of the filters used in the search process has been conducted.

According to the filters applied in the search engines, 79% (22/28) of papers resulted from the “emergency” AND “app” filter and 17% (5/28) resulted from the “emergency” AND “mHealth” filter. Moreover, 4% (1/28) of the papers resulted from the “emergency” AND “eHealth” filter. The “eEmergency” filter did not return relevant results.

**Table 8:** Categorization of apps (Android and iOS).

Categorization	Number of apps
Health and Wellness	73
Medicine	68
Tools	36
Lifestyle	26
Travel and Guides	23
Communication	18
Social	16
Business	14
News and Magazines	14
Maps and Navigation	13
Education	11
Productivity	8
Entertainment	4

**Table 9:** Categorization of apps by filter (Android and iOS).

Categorization	Number of apps
Emergency OR emergencia	89
Blood donation OR donación de sangre	79
SOS	56
Alert OR alerta	34
112	24
Safety OR seguridad	23
Disaster OR desastre	10
eEmergency	9

## Discussion

### Principal Findings

The 28 obtained papers were categorized by the authors into the following six different groups: (1) prehospital medical care; (2) apps for disaster management; (3) warning systems for emergency services and medical services; (4) automobile circulation control; (5) communication between medical staff; and (6) apps for blood donation. The classification of the analyzed studies is presented in Table 10.

Warning systems for emergency services and medical services led, with 39% (11/28) of the obtained publications. These systems are usually apps in which medical services are notified with the press of a button. Followed by this, we have the group of apps for disaster management, owing to the problems that exist with different natural disasters in Eastern countries (6/28 [21%] of the obtained publications). These two groups had a higher percentage than the rest of the groups. Communication between medical staff is critical for the success of emergency care services. Effective and efficient methods of communication involving health care staff play major roles in improving global health care services. Apps for blood donation are also critical, since in emergency scenarios, the availability of the correct blood type for a patient is crucial for the recovery process.

Prehospital care should be highlighted to include prehospital medical care methods, as well as automobile circulation control for the circulation of medical vehicles since it directly influences an improvement in prehospital medical care by improving the time it takes for a medical vehicle to care for patients.

mHealth has great potential, as it can provide citizens with the necessary means to manage their health and stay healthy longer. Consequently, people can improve the quality of health care and patient comfort, and help health professionals in their work.

The search for mHealth solutions can contribute to the development of modern, efficient, and sustainable health systems. It is also expected to reduce costly visits to the hospital, help citizens to take charge of their state of health and well-being, and promote health focused on prevention rather than cure. Furthermore, it is an excellent opportunity for the flourishing app sector and entrepreneurs.

This literature review mentioned the relevant usage of mobile apps in the health emergency domain. In addition, this paper stated the need to investigate the realization of more studies on prehospital care. Recent studies are focused on mHealth technology, and they leave aside the different branches that may arise from this technology.

Wearable devices are becoming increasingly relevant not only in applications in the health sector, but also in global mobile telecommunication. The applications can be notified through a mobile device, such as a smartphone or tablet and a wearable device.

Considering the extensive inclusion of 5G technology, mobile communication technology can be assumed to be experiencing a breakthrough, since we can send more information in seconds and with less energy consumption. In addition, the network coverage offered by mobile communication technologies can help to reach remote areas such as mountains. Moreover, at present, mobile networks offer high data transfer rates, which enable remote surgery tasks. Finally, given the increase in the use of mobile devices worldwide, we found a large number of apps.

There was greater availability of apps for Android than for iOS. Furthermore, Android apps were free compared with iOS apps (73/132 [55.5%] were paid). Considering the large number of apps found in the category of Medicine in this study, we can conclude that mobile apps are mainly introduced in this area. Moreover, the category of Health and Wellness involved even more mobile apps. Even though mobile technology has increased, there is much growth in the field of health emergencies and mHealth.

After the completion of this work, three essential aspects are planned as future lines of research. Owing to the tremendous digital transformation experienced by both industry and society, they increasingly require the use of a highly organized infrastructure on the network, that is, on the internet, and the loading or unloading of large amounts of data, which, in the case of health, is of utmost importance. Therefore, the three most relevant technologies that will be increasingly relevant owing to both the demand for cloud services and large volumes of data, and the search for the solvency of vulnerabilities in data management are cybersecurity, big data, and cloud computing. In addition, wearable devices are a critical matter of study in the mHealth app space. Finally, internet of things and smart sensor communication are becoming increasingly widespread and are crucial for enhanced telemedicine.

**Table 10:** Distribution of the studies per category.

Category	References	Number of studies
I. Prehospital medical care	[14,26,32]	3
II. Apps for disaster management	[15,18,31,33,38,39]	6
III. Warning systems for emergency and medical services	[13,16,17,21,22,28-30,35-37]	11
IV. Automobile circulation control	[27,40]	2
V. Communication between medical staff	[19,25,34]	3
VI. Apps for blood donation	[20,23,24]	3

### Conclusion

We conducted an analysis of the current status of mHealth technologies and apps for medical emergencies. The PRISMA methodology was used in this review. First, the available literature of the previous 10 years (2009-2019) was analyzed. Second, a review of mobile apps in the two common virtual storage platforms (Google Play Store and Apple App Store) was carried out. The Google Play Store and Apple App Store are for the Android and iOS operating systems, respectively.

In total, 28 papers in the field of medical emergencies were included. These studies were categorized into six different groups. Overall, 39% (11/28) of the included studies were related to warning systems for emergency services and 21% (6/28) were associated with disaster management apps.

In total, 324 mobile apps were found, with 59.3% (n=192) identified in the Google Play Store and 40.7% (n=132) identified in the Apple App Store. All identified mobile apps in the Google Play Store were free, and in the Apple App Store, 55.5% (73/132) of the identified apps were paid, with the price ranging from US $0.89 to US $5.99.

## Abbreviations

mHealth: mobile health
PDA: personal digital assistant device
PRISMA: Preferred Reporting Items for Systematic Reviews and Meta-Analyses
QUOROM: Quality of Reporting of Meta-Analysis

## References

[1] (2019). Encuesta sobre Equipamiento y Uso de Tecnologías de Información y Comunicación en los Hogares. Instituto Nacional de Estadística.

[2] Fernandez-Luque L, Labarta JI, Palmer E, Koledova E (2020). Content Analysis of Apps for Growth Monitoring and Growth Hormone Treatment: Systematic Search in the Android App Store. JMIR Mhealth Uhealth.

[3] Gazdecki A 9 Mobile Technology Trends For 2017 (Infographic). BiznessApps.

[4] La Sociedad de la Información en España 2016. Fundación Telefónica.

[5] Martinez-Millana A, Jarones E, Fernandez-Llatas C, Hartvigsen G, Traver V (2018). App Features for Type 1 Diabetes Support and Patient Empowerment: Systematic Literature Review and Benchmark Comparison. JMIR Mhealth Uhealth.

[6] Thomas L Global Mobile Health Market Size, Share, Growth, Trends, and Forecast, 2016-2022. America News Hour.

[7] Gaziel-Yablowitz M, Schwartz DG (2018). A Review and Assessment Framework for Mobile-Based Emergency Intervention Apps. ACM Comput. Surv.

[8] Franco-Martín MA, Muñoz-Sánchez JL, Sainz-de-Abajo B, Castillo-Sánchez G, Hamrioui S, de la Torre-Díez I (2018). A Systematic Literature Review of Technologies for Suicidal Behavior Prevention. J Med Syst.

[9] Moher D, Cook D, Eastwood S, Olkin I, Rennie D, Stroup D (2000). Improving the Quality of Reports of Meta-Analyses of Randomised Controlled Trials: The QUOROM Statement. Onkologie.

[10] Moher D, Liberati A, Tetzlaff J, Altman DG, PRISMA Group (2009). Preferred reporting items for systematic reviews and meta-analyses: the PRISMA statement. Ann Intern Med.

[11] Góngora Alonso S, Hamrioui S, de la Torre Díez I, Motta Cruz E, López-Coronado M, Franco M (2019). Social Robots for People with Aging and Dementia: A Systematic Review of Literature. Telemed J E Health.

[12] de la Torre Díez I, Alonso SG, Hamrioui S, López-Coronado M, Cruz EM (2018). Systematic Review about QoS and QoE in Telemedicine and eHealth Services and Applications. J Med Syst.

[13] Huffman A (2015). With the Proliferation of Mobile Medical Apps, Which Ones Work Best in the Emergency Department?. Annals of Emergency Medicine.

[14] Sarlan A, Xiong FK, Ahmad R, Ahmad WF, Bhattacharyya E (2015). Pre-hospital emergency notification system.

[15] Li P, Miyazaki T, Wang K, Guo S, Zhuang W (2017). Vehicle-assist resilient information and network system for disaster management. IEEE Trans. Emerg. Topics Comput.

[16] Bhati N (2017). MHealth based ubiquitous fall detection for elderly people.

[17] Almuraykhi KM, Akhlaq M (2019). STLS: Smart Traffic Lights System for Emergency Response Vehicles.

[18] Sun BT, Hu HB, Chen XZ (2019). State of the Earthquake Field Disaster Investigation Information Service System.

[19] Johnston MJ, King D, Arora S, Behar N, Athanasiou T, Sevdalis N, Darzi A (2015). Smartphones let surgeons know WhatsApp: an analysis of communication in emergency surgical teams. Am J Surg.

[20] Fahim M, Cebe HI, Rasheed J, Kiani F (2016). mHealth: Blood donation application using android smartphone.

[21] Tripti NF, Farhad A, Iqbal W, Zaman HU (2019). SaveMe: A Crime Deterrent Personal Safety Android App with a Bluetooth Connected Hardware Switch.

[22] Pughazendi N, Sathish Kumar R, Karthikeyan A, Seshan PV (2018). Click away emergency aid scheme by means of intelligent situation assessment.

[23] Annish Brislin MR, Albert Mayan J, Aroul Canessane R, Anish Hamlin MR (2017). Blood donation and life saver app.

[24] Pohandulkar SS, Khandelwal CS (2018). Blood Bank App using Raspberry PI.

[25] Yamada KC, Inoue S, Sakamoto Y (2015). An effective support system of emergency medical services with tablet computers. JMIR Mhealth Uhealth.

[26] Kang JJ, Venkatraman S (2018). An Integrated mHealth and Vehicular Sensor Based Alarm System Emergency Alarm Notification System for Long Distance Drivers using Smart Devices and Cloud Networks.

[27] Satya RL, Kaviya R, Valarmathi R (2018). Intelligent Crash Detection and Emergency communication system for Two Wheelers.

[28] Gómez B, Juiz C (2013). Integration of Emergency Web App for accessing the emergency services by mobile phones.

[29] Nair MB, Kumar SR, Kishore NA, Mohan N, Anudev J (2018). Instantaneous feedback pedometer with emergency GPS tracker.

[30] Jen YY, Jason Chang SK (2016). Information and communication technologies for enhanced Emergency Management in Taiwan High Speed Rail.

[31] Jayapravitra Y, Prasertsil W, Limim N, Phatthanacharoensuk I, Songsinviboon T (2017). iEMS1669: An innovative Med Alert App for Thai Emergency Medical System.

[32] Fleshman MA, Argueta IJ, Austin CA, Lee HH, Moyer EJ, Gerling GJ (2016). Facilitating the collection and dissemination of patient care information for emergency medical personnel.

[33] Depari A, Flammini A, Fogli D, Magrino P (2018). Indoor Localization for Evacuation Management in Emergency Scenarios.

[34] Gulacti U, Lok U, Hatipoglu S, Polat H (2016). An Analysis of WhatsApp Usage for Communication Between Consulting and Emergency Physicians. J Med Syst.

[35] Elsaadany A, Sedky A, Elkholy N (2017). A triggering mechanism for end-to-end IoT eHealth system with connected ambulance vehicles.

[36] Psaila G, Scandurra P, Rovelli S, Mazzucchelli E, Taiocchi M (2014). A Mobile/Cloud Emergency Response Application for Indoor Assisted Living.

[37] Edillo SB, Garrote PJ, Domingo LC, Malapit AG, Fabito BS (2018). A mobile based emergency reporting application for the Philippine National Police Emergency Hotline 911: A case for the development of i911.

[38] Layek AK, Pal A, Saha R, Mandal S (2018). DETSApp: An App for Disaster Event Tweets Summarization using Images Posted on Twitter.

[39] Li L, Ulaganathan MN (2017). Design and development of a crowdsourcing mobile app for disaster response.

[40] Noei S, Santana H, Sargolzaei A, Noei M (2014). Reducing Traffic Congestion Using Geo-fence Technology: Application for Emergency Car. EMASC '14: Proceedings of the 1st International Workshop on Emerging Multimedia Applications and Services for Smart Cities.

